# The Importance of Excisional Biopsy in Nodular Lymphocyte-Predominant Hodgkin’s Lymphoma

**DOI:** 10.7759/cureus.40225

**Published:** 2023-06-10

**Authors:** Angel Juarez, Kevin Parza, Lidice Galindo, Mohamed Faris

**Affiliations:** 1 Internal Medicine, Grand Strand Medical Center, Myrtle Beach, USA

**Keywords:** non-hodgkin’s lymphoma, hodgkin’s lymphoma, nodular lymphocyte-predominant hodgkin’s lymphoma, supraclavicular lymphadenopathy, hematology-oncology, excisional tissue biopsy

## Abstract

Hodgkin’s lymphoma (HL) is a malignancy that is typically B-cell in origin. HL can be further classified into classical HL and nodular lymphocyte-predominant HL (NLPHL). NLPHL is a rare lymphoma. It commonly presents locally with palpable firm lymphadenopathy or mediastinal mass seen on chest imaging. Some patients may have B symptoms (fever, night sweats, and unintentional weight loss), splenomegaly, and hepatomegaly. We describe a case of NLPHL in a 32-year-old male with classical findings of this rare class of HL.

## Introduction

Nodular lymphocyte-predominant Hodgkin’s lymphoma (NLPHL) is a rare lymphoma with an incidence of 0.1 to 0.2/100,000 per year [1). NLPHL represents approximately 5% of lymphomas and typically affects children and young adults. It usually presents with painless lymphadenopathy. Cervical lymph nodes are often seen in 60-70% of patients, axillary lymph nodes in 25-35% of patients, and at times inguinal lymph nodes in 8-15% of patients [1]. As seen in this case, patients may also present with splenomegaly or hepatomegaly. NLPHL has the distinctive features of lymphocyte-predominant (LP) cells, also known as popcorn cells. NLPHL can be difficult to diagnose as this lymphoma may contain infrequent tumor cells in a background of non-neoplastic cells. If not diagnosed, some cases can progress to a more aggressive lymphoma such as diffuse large B-cell lymphoma [1,2]. This case highlights the importance of adhering to the gold standard of an excisional biopsy, as without it, this rare Hodgkin’s lymphoma (HL) can be difficult to diagnose.

## Case presentation

We present the case of a 32-year-old male with progressive left upper quadrant pain and worsening fatigue with night sweats. He self-reported that in the previous year, he had experienced weight loss of an unspecified amount and constant night sweats requiring multiple changes of clothes throughout the night. He also noted early satiety, with decreased oral intake due to his abdominal pain. He was prompted to seek medical attention as the pain became intolerable. Physical examination on initial encounter showed clear breath sounds bilaterally, a regular heart rhythm, and significant diffuse lymphadenopathy of the anterior and posterior cervical chain bilaterally which were non-tender. Abdominal examination was significantly tender to light touch in the left upper quadrant with a palpable spleen estimated 10 cm below the left costal margin. Vital signs on presentation were as follows: temperature 100.8°F, pulse 98 beats per minute, respiratory rate 18 breaths per minute, and blood pressure 99/63 mmHg. Initial labs are presented in Table 1.

**Table 1:** Initial laboratory results on admission. WBC: white blood cells; Hgb: hemoglobin; HCT: hematocrit; MCV: mean corpuscular volume; RDW: red cell distribution width; PLT: platelets; BUN: blood urea nitrogen; eGFR: estimated glomerular filtration rate; AST: aspartate aminotransferase; ALT: alanine transaminase; LDH: lactate dehydrogenase; INR: international normalized ratio; PT: prothrombin time; and PTT: partial thromboplastin time

Lab	Results	Normal range
Complete blood count		
WBC	5.3 K/mm^3^	3.7–10.1 K/mm^3^
Hgb	8.4 g/dL	14.0–16.4 g/dL
HCT	26.0%	40.0–47.2%
MCV	77.8 fL	81.8–94.6 fL
RDW	20.7%	11.6–14.0%
PLT count	183 K/mm^3^	150–400 K/mm^3^
Chemistry panel		
Sodium	138 mmol/L	136–145 mmol/L
Potassium	4.0 mmol/L	3.5–5.1 mmol/L
Chloride	99 mmol/L	98–107 mmol/L
Carbon dioxide	32 mmol/L	21–32 mmol/L
Anion gap	7.0 mEq/L	3.0–11.0 mEq/L
BUN	16 mg/dL	7–18 mg/dL
Creatinine	0.81 mg/dl	0.6–1.3 mg/dL
eGFR (CKD-EPI)	>60	≥60
Glucose	105 mg/dL	74–106 mg/dL
Calcium	9.1 mg/dL	8.5–10.1 mg/dL
AST	18 U/L	11–38 U/L
ALT	33 U/L	10–47 U/L
Total protein	8.1 g/dL	6.4–8.2 g/dL
Albumin	3.6 g/dL	3.5–5.0 g/dL
Lactic acid	1.0 mmol/L	0.7–2.1 mmol/L
LDH	139 U/L	100–190 U/L
Coagulation panel		
INR	1.66	0.9–1.1
PT	19.1 seconds	9.8–13.9 seconds
PTT	34.5 seconds	24.9–37.9 seconds

On further review, the patient had a previous hospital admission one year prior for abdominal pain with similar B symptoms. CT imaging at that time revealed severe enlargement of the spleen, multiple enlarged upper abdominal retroperitoneal lymph nodes, and right supraclavicular lymphadenopathy. During that admission, he underwent a right cervical core needle biopsy revealing a reactive lymph node with no evidence of lymphoproliferative disorder. He was discharged and seen as an outpatient by radiation oncology for three treatments of radiation therapy for his splenomegaly.

**Figure 1:**
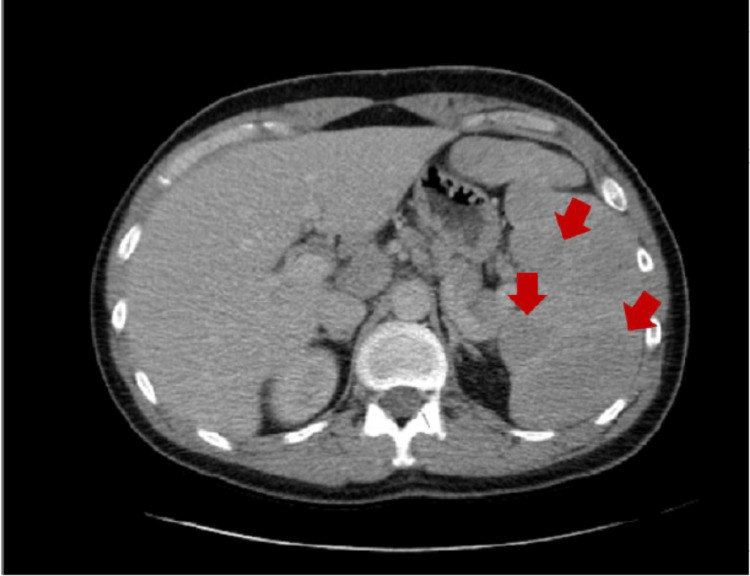
CT of the abdomen with contrast. This is a transverse CT cross-section showing an enlarged spleen with multiple hypoattenuating lesions (red arrows).

**Figure 2:**
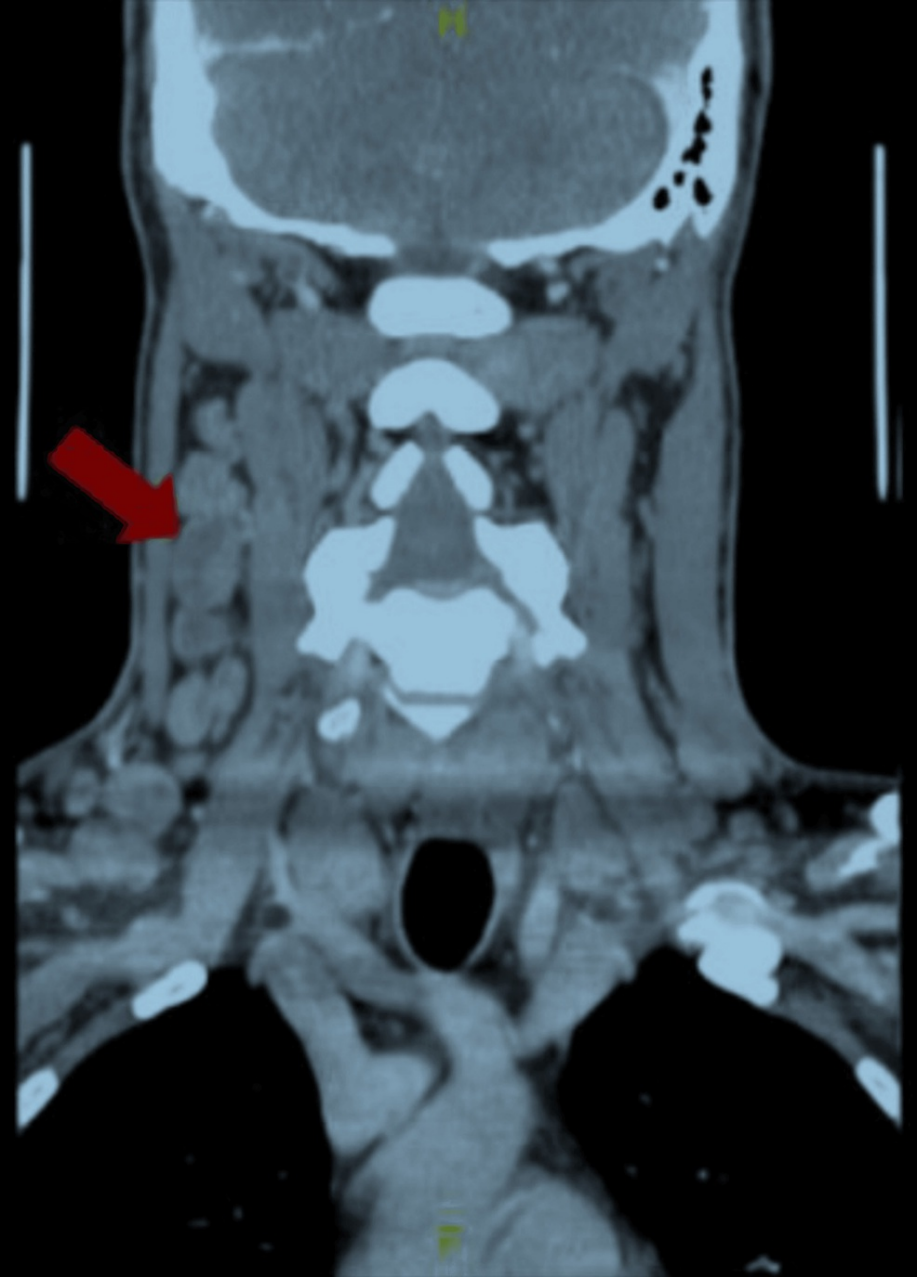
CT of the neck. This is a frontal plan of the patient’s CT of the neck showing multiple cervical adenopathy most pronounced on the right side of the neck (red arrows).

On further workup, human immunodeficiency virus (HIV), toxoplasmosis, antinuclear antibody (ANA), Mantoux test, cytomegalovirus, and hepatitis A, B, and C were negative. Epstein-Barr virus IgG was positive. IgG, IgA, and IgM were within normal limits. Interleukin 6 (IL-6) was 26.2 pg/mL (normal values = 0.0-13.0 pg/mL). Repeat abdominal and chest CT with contrast showed marked splenomegaly, measuring approximately 16.7 cm with multiple hypoattenuating lesions in the spleen with innumerable focal lesions increasing in the left upper quadrant, periportal, and retroperitoneal lymphadenopathy (Figure 1). CT of the chest did not show any cardiopulmonary process, no mediastinal mass, or lung nodules. CT of the neck showed supraclavicular and cervical adenopathy, most pronounced on the right (Figure 2).

**Figure 3:**
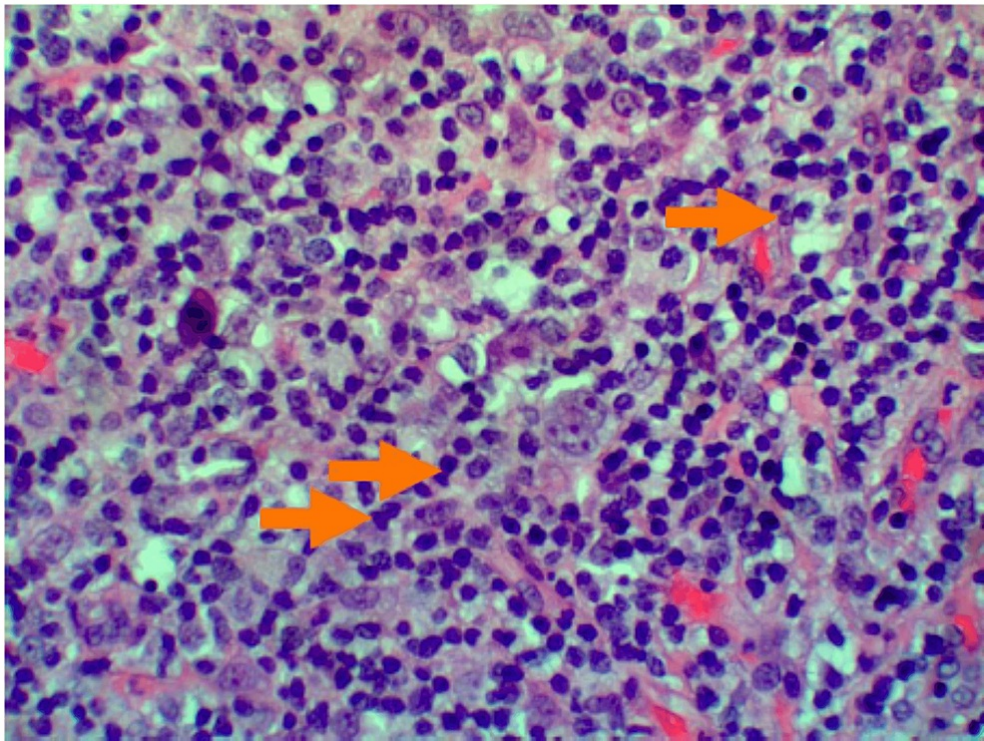
Hematoxylin and eosin stain. Hematoxylin and eosin staining in high-power view demonstrating atypical lymphoid cells with prominent multinuclear lobation and nucleoli (orange arrows).

**Figure 4:**
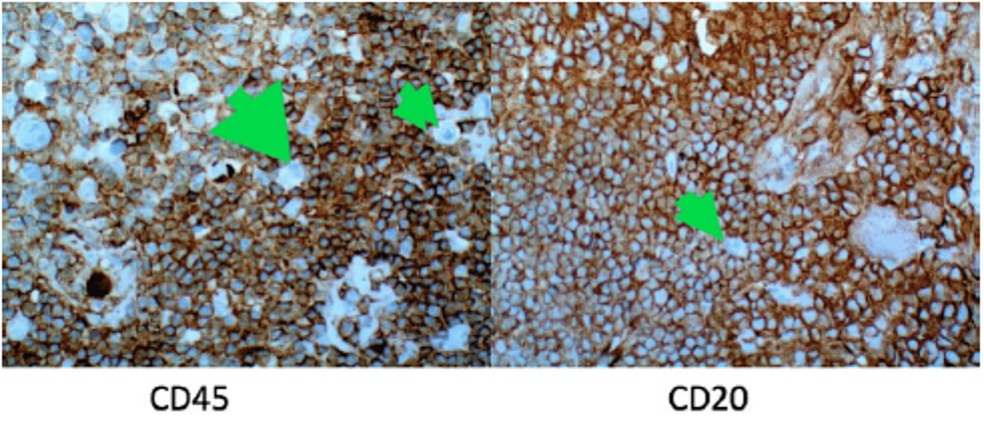
Immunohistochemistry stains. These are slides on immunohistochemistry staining for CD45 (left) and CD20 (right). Green arrows show large atypical cells.

**Figure 5:**
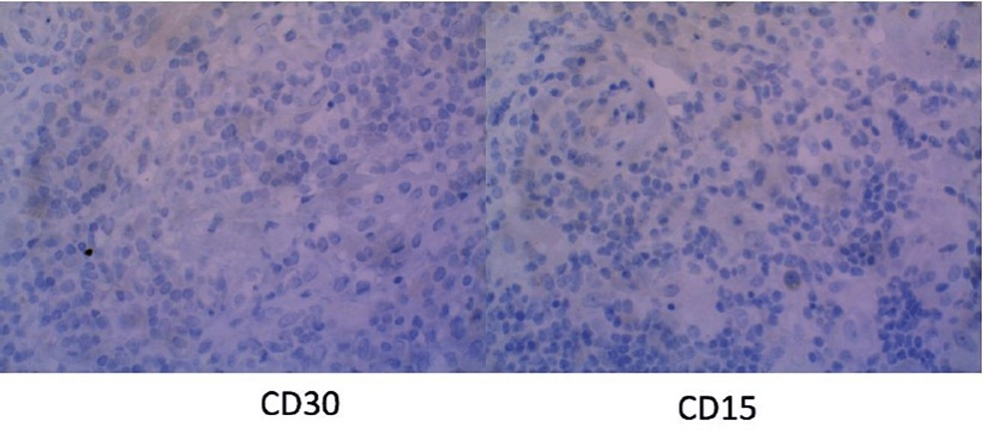
Immunohistochemistry stains. The above slides are immunohistochemistry staining of CD30 and CD15 which were negative in this patient.

Shortly thereafter, the patient underwent a right posterior cervical node excisional biopsy. The results were pertinent for an enlarged lymph node with atypical lymphoid cells (Figure 3). Immunohistochemistry (IHC) and flow cytometry immunophenotyping were also performed which demonstrated CD20 positivity with a subset showing positivity for CD45 (Figure 4). CD30 and CD15 were also tested and were unremarkable (Figure 5). In addition, paired box 5, epithelial membrane antigen, B-cell lymphoma 6, and multiple myeloma 1 were not observed. These findings were consistent with a final diagnosis of NLPHL.

**Figure 6:**
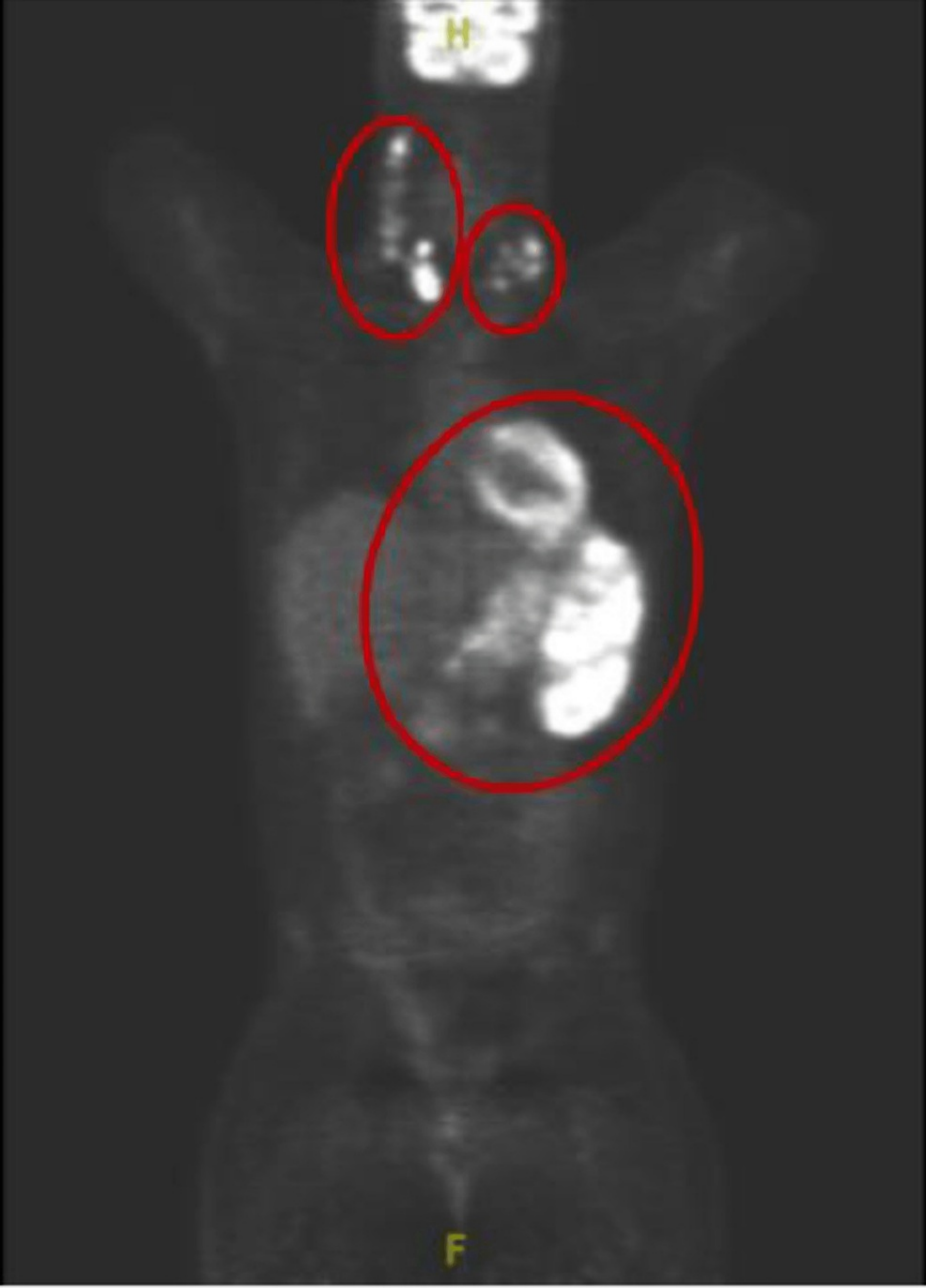
Positron emission tomography. Coronal plane image of positron emission tomography/computed tomography scan taken before treatment. The image shows adenopathy activity above and below the diaphragm with splenic infiltration encased in red circles.

The patient was discharged home to begin treatment at an outpatient facility. Positron emission tomography/computed tomography (PET/CT) done before initiation showed adenopathy activity above and below the diaphragm with splenic infiltration (Figure 6).

## Discussion

HL is a malignancy that is typically B-cell in origin. The World Health Organization (WHO) classifies HL into two types, namely, classical HL and NLPHL. Classical HL can be further subdivided into nodular sclerosis, mixed cellularity, lymphocyte-depleted, and lymphocyte-rich. Our focus is on NLPHL, a rare lymphoma with an incidence of 0.1 to 0.2/100,000 per year [1,3].

NLPHL represents approximately 5% of HLs and typically affects children and young adults. It usually presents with painless lymphadenopathy. Patients may also present with splenomegaly or hepatomegaly with B symptoms. NLPHL can be difficult to diagnose as it may contain infrequent tumor cells in a background of non-neoplastic cells. The presence of LP cells is a prerequisite for the diagnosis of NLPHL. In general, classical HLs are aggressive while NLPHL has an indolent biology. However, if not properly treated, some cases can progress to a more aggressive lymphoma such as diffuse large B-cell lymphoma [1,2].

The exact causes of HL are unknown. Nevertheless, several risk factors have been associated with this pathology. Epsietin-Barr virus has a strong association with HL, which was also seen in our patient. Other associations are patients with immunodeficiency such as organ or cell transplantation, immunosuppressants, HIV infection, or chemotherapy. There is also an association with autoimmune diseases such as rheumatoid arthritis and sarcoidosis.

Suspicion of this disease process begins with a medical history and clinical features of B symptoms (fever, night sweats, and weight loss), and localization of lymph nodes. A complete blood count may show anemia, with an elevated or decreased white blood cell count. Serum chemistry usually shows an elevated lactate dehydrogenase. Inflammatory markers, such as erythrocyte sedimentation rate or C-reactive protein, can also be elevated and may serve as useful lab markers of disease response [3,4]. IL-6 is a potent immunomodulatory cytokine that may have significance in several malignancies including HL. In a small cohort study, patients with HL had an elevated serum IL-6 level noted pre-therapy with a decrease in levels to normal values at the time of remission. However, the role of IL-6 remains unclear [5,6].

Lymph node excisional biopsy of the involved nodes is preferred and the gold standard to establish a definitive diagnosis. Occasionally, core biopsies may be adequate but often fall short as neoplastic cells may be missed in these specimens. Fine-needle aspirates rarely reveal architecture and may be insufficient for new diagnosis [3,4]. LP cells express CD20, CD79a, and CD45. Unlike in classical HL, these LP cells are negative for CD15 and CD30, as seen in our case. Once diagnosed, the patient should undergo a staging evaluation using the Lugano criteria before treatment. This includes clinical evaluation, laboratory studies, and imaging modalities such as PET and/or CT. Bone marrow biopsy is not routinely performed unless there are unexplained cytopenias or suspicion of bone marrow involvement.

Treatment usually consists of chemotherapy. Radiation is not routinely used; however, occasionally, it may be used for large lymph nodes or lymph nodes that are symptomatic. The most widely used therapy approach is adriamycin, bleomycin, vinblastine, and dacarbazine, especially in the early stages. However, recently, rituximab in addition to cyclophosphamide, doxorubicin, vincristine, and prednisone, followed by optional adjuvant radiation therapy in newly diagnosed advanced NLPHL has also been used. In addition, there is less toxicity compared to bleomycin, etoposide, doxorubicin, cyclophosphamide, vincristine procarbazine, and prednisone regimen; therefore, the former has a more favorable risk-benefit ratio [7,8].

Outcomes are favorable for early-stage disease compared to advanced stages of NLPHL. In a retrospective analysis of 394 patients, complete remission was achieved in 92% in the early stages and 77% in individuals with advanced diseases [9]. Even after remission, patients should be monitored for possible relapse.

## Conclusions

This is a case of a 32-year-old male with newly diagnosed NLPHL. This subtype of HL is rare and at times difficult to diagnose due to infrequent tumor cells seen on biopsy. In this case, there was a delay in treatment as there was an inadequate amount of tissue in the fine-needle biopsy. Physicians need to be aware and mindful of this as excisional biopsy is the gold standard for diagnosis. Fortunately, there is a good prognosis for this disease process when properly diagnosed and treated.
